# Vestibular Loss and Balance Training Cause Similar Changes in Human Cerebral White Matter Fractional Anisotropy

**DOI:** 10.1371/journal.pone.0095666

**Published:** 2014-04-28

**Authors:** Nadine Hummel, Katharina Hüfner, Thomas Stephan, Jennifer Linn, Olympia Kremmyda, Thomas Brandt, Virginia L. Flanagin

**Affiliations:** 1 German Center for Vertigo and Balance Disorders, University Hospital Munich, Campus Grosshadern, Munich, Germany; 2 Graduate School of Systemic Neurosciences, Ludwig-Maximilians University, Planegg-Martinsried, Germany; 3 Department of Neurology, University Hospital Munich, Campus Grosshadern, Munich, Germany; 4 Institute for Clinical Neurosciences, University Hospital Munich, Campus Grosshadern, Munich, Germany; 5 Department of Neuroradiology, University Hospital Munich, Campus Grosshadern, Munich, Germany; University of Minnesota, United States of America

## Abstract

Patients with bilateral vestibular loss suffer from severe balance deficits during normal everyday movements. Ballet dancers, figure skaters, or slackliners, in contrast, are extraordinarily well trained in maintaining balance for the extreme balance situations that they are exposed to. Both training and disease can lead to changes in the diffusion properties of white matter that are related to skill level or disease progression respectively. In this study, we used diffusion tensor imaging (DTI) to compare white matter diffusivity between these two study groups and their age- and sex-matched controls. We found that vestibular patients and balance-trained subjects show a reduction of fractional anisotropy in similar white matter tracts, due to a relative increase in radial diffusivity (perpendicular to the main diffusion direction). Reduced fractional anisotropy was not only found in sensory and motor areas, but in a widespread network including long-range connections, limbic and association pathways. The reduced fractional anisotropy did not correlate with any cognitive, disease-related or skill-related factors. The similarity in FA between the two study groups, together with the absence of a relationship between skill or disease factors and white matter changes, suggests a common mechanism for these white matter differences. We propose that both study groups must exert increased effort to meet their respective usual balance requirements. Since balance training has been shown to effectively reduce the symptoms of vestibular failure, the changes in white matter shown here may represent a neuronal mechanism for rehabilitation.

## Introduction

Peripheral bilateral vestibular failure is a disorder of various etiologies characterized by a lack of vestibular input due to vestibular nerve or hair cell damage. Patients suffer from severe difficulties in maintaining balance, causing unsteadiness of gait and a high risk of falls. Symptoms can also include dizziness, nausea and oscillopsia, as well as cognitive impairments, although the causal relationship between vestibular failure and cognitive deficits is still unclear [1], [2]. The non-invasive method of vestibular rehabilitation therapy, which comprises different balance tasks and exercises, is used to treat symptoms of vestibular failure [3], [4].

Various sports also put a high demand on the ability to maintain balance, and require the use and interpretation of vestibular information to correctly perform e.g. a dancer's pirouette, without a sense of vertigo. Ballet dancers can reduce their vestibular-ocular reflex (VOR) in response to spinning [5], [6] suggesting that their vestibular system is affected by the training required to perform their sport. Slacklining, a relatively new balance sport, was also shown to decrease reflectory muscle reactions and have a positive influence on postural control [7], another behavior where vestibular information is quite important.

Both training [8] and disease [1] have been shown to lead to significant changes in brain structure, or plasticity. Vestibular failure in humans causes volumetric decreases in gray matter structures involved in vestibular processing such as the thalamus, parietal-temporal regions, area MT/V5 and the hippocampus [1], [9]. Ballet training has a reductive effect on grey and white matter volume and on fractional anisotropy within frontal and motor areas [10]. Figure skating and slacklining also show structural modifications in the brain [11]. Each of these balance sports have different requirements in terms of interpreting sensory-motor information, but all require a reinterpretation of vestibular information, which is also necessary after bilateral vestibular loss. However, a comparison of the effects of vestibular loss and professional balance training on the brain structure has yet to be done. This comparison may prove useful in understanding the mechanisms underlying vestibular disease, training and rehabilitation.

In this study, we investigated the differential effects of increased and decreased balance ability on white matter plasticity using diffusion tensor imaging (DTI). This method can be used to detect microstructural changes in white matter by measuring the water diffusion directionality in nerve fibers [12]. The fractional anisotropy (FA) of the diffusion tensor can be separated into axial (AD, parallel to nerve fibers) and radial diffusivity (RD, perpendicular to nerve fibers) components. Here, we compared the FA, RD and AD of patients with chronic bilateral vestibular failure, healthy balance trained subjects, such as ballet dancers, figure skaters and slackliners and their respective control groups to identify 1) plastic white matter changes that are related to vestibular input in general and 2) overlapping regions of white matter restructuring in both disease and training.

## Methods

### Ethics statement

All subjects gave written informed consent to participate in the study, which was approved by the ethics committee of the medical faculty of the Ludwig Maximilians University and performed in accordance with The Code of Ethics of the World Medical Association (Declaration of Helsinki) for experiments involving humans. The use of minors was accepted by the ethics committee and we obtained written informed consent from the parents or guardians of the subjects, as well as written informed consent from the subjects that were under age.

### Subjects

13 patients with bilateral vestibular failure (BVF, six females, mean age: 65.38, range: 44–86), their healthy controls (BC, n = 13, five females, mean age: 63.54, range: 42–80), 18 balance trained persons, including five ballet dancers, five figure skaters, one person doing both, ballet dancing and figure skating and seven slackliners (T, eight females, mean age: 25, range 16–43) and their healthy controls (TC, n = 17, ten females, mean age: 26.18, range 21–39) participated in the study. Control subjects were matched for age and sex. TC were additionally matched for the amount of leisure sports. In other words, the controls had a certain level of physical activity, i.e. they performed leisure sports, like swimming, jogging, dancing etc., that the trained group also did in addition to their balance sport. The range of leisure sports and the overall amount of additional physical activity were comparable between the two groups. All healthy participants had no history of neurological disorders and no history of dizziness or vestibular disorders. Head impulse tests were done on all subjects to check vestibular function. Ballet dancers and figure skaters had been training for 11–34 years (16.4±6.88 years; mean ± SD), slackliners for 1–8 years (2.79±2.53 years; mean ± SD). All members of group T trained at least two hours a week, except for one dancer who had a foot injury at the time of measurement. Further details about training load, i.e. the current amount of hours spent training per week and the overall training experience can be found in Table 1. A heterogeneous balance trained group was purposefully chosen to look for overall effects of balance training, independent of the specific type of sport done.

**Table 1:** Characteristics of subjects trained in balance sports.

ID	Sex	Age	Training type	Training (yrs)	Current training (h/week)	Experience^a^
T01	f	40	Ballet	34	3	2040
T02	f	19	Ballet	11	48	528
T03	m	26	Ballet	19	30	570
T04	f	23	Ballet	12	7.5	90
T05	m	29	Ballet	20	42	840
T06	m	25	Figure skating	16	6	224
T07	f	17	Figure skating	13	0	130
T08	m	17	Figure skating	13	7.5	97.5
T09	f	16	Figure skating/ballet	13/12	3	130
T10	f	16	Figure skating	14	10	140
T11	f	17	Figure skating	12	10	96
T12	m	28	Slacklining	4	6	24
T13	m	24	Slacklining	1	10	8
T14	m	43	Slacklining	1	2	6
T15	f	38	Slacklining	8	6	48
T16	m	21	Slacklining	1.5	10	15
T17	m	27	Slacklining	1.5	6	9
T18	m	24	Slacklining	2.5	3	5

a Experience was calculated by multiplying the hours of training per week averaged over the past year by the number of years the individual had been practicing the activity.

The patients (Table 2) in this study were recruited from the Interdisciplinary Dizziness Clinic of the German Center for Vertigo and Balance Disorders, Munich and met the following inclusion criteria: 1) bilateral pathological head impulse test and 2) bilateral reduced (mean slow phase eye velocity ≤6°/s) or absent responsiveness in the bithermal caloric irrigation 3) no clinical signs indicating cerebellar dysfunction and 4) no additional neurological diseases. All patients suffered from chronic bilateral vestibular hypofunction, i.e. at the time of measurement, they have been living with the disease for at least two years. None of the patients had regularly undergone vestibular rehabilitation therapy at the time of measurement. One patient had a mean slow phase eye velocity of 6.4°/s which marginally exceeds the lower limit of 6.0°/s. We decided to include this patient because all other inclusion criteria were matched and the limit violation was only small. Heterogeneity of disease etiology was deliberate to ensure that our findings are most likely due to a decrease or lack of vestibular sensory input and not the result of other unforeseen factors related to a specific disease.

**Table 2:** Characteristics of patients with bilateral vestibular failure.

ID	Sex	Age	Etiology	Time since onset (yrs)	Caloric mean SPEV^a^ (°/s)
BVF01	m	79	Aminoglycosides	10	0.875
BVF02	f	86	Meningitis	69	0
BVF03	m	58	Idiopathic	4	0
BVF04	f	67	Borreliosis	14	1.175
BVF05	m	58	Traumatic	14	4.5
BVF06	f	68	Autoimmune	12	1.575
BVF07	m	65	Meningitis	35	0
BVF08	m	63	Idiopathic	2	1.75
BVF09	f	44	Idiopathic/familial	10	0
BVF10	m	61	Idiopathic	2	0
BVF11	f	66	Ménière's disease	13	6.4
BVF12	f	59	Idiopathic	2	5.25
BVF13	m	78	Idiopathic	5	2

a SPEV = slow phase eye velocity.

### Diffusion-weighted image acquisition

Image acquisition was performed on a 3T MRI Scanner (*Signa HDx, GE Healthcare, Milwaukee, USA*) with a standard 8-channel head coil. A diffusion weighted single shot spin-echo sequence (repetition time 10000 ms, echo time 84 ms, b-value = 1000 s/mm^2^, 20 directions, 256×256 matrix, 2.5 mm slice thickness, 40 slices, FOV 25 cm, with one b0 image without diffusion weighting) was collected along with a high-resolution T1-weighted anatomical sequence (0.8 mm isotropic voxel size).

### Image processing and data analysis

All preprocessing and whole brain analyses were carried out with FMRIB Software Library FSL, version 4.1.8 [13] following the protocol described in Smith et al. (2007) [14]. Diffusion data from every subject was corrected for head motion and eddy current effects using the eddy current correction tool of the FMRIB's Diffusion Toolbox (FDT). Brain images were extracted using the brain extraction tool (BET) [15]. Diffusion tensors were fitted with the FDT's dtifit tool. Voxelwise analysis of the data was carried out using TBSS (Tract-Based Spatial Statistics) [16] in FSL. All subjects' FA data were aligned into a common space (defined by the FMRIB58_FA template in FSL) using the nonlinear registration tool FNIRT [17], which uses a b-spline representation of the registration warp field [18]. Next, single subject FA images were averaged. This mean FA image was thinned using a threshold of 0.2 to create a mean FA skeleton, representing the centers of all white matter tracts common to the group. Each subject's aligned FA data was then projected onto this skeleton and the resulting data fed into voxelwise cross-subject statistics.

### Statistics

We conducted statistical analyses to test for differences in FA between the four groups (BVF, T, BC, TC). We identified the source of the FA differences by further determining radial and axial diffusion components (RD and AD respectively). Additionally, we conducted correlation analyses to identify the effects of age and measures of cognition, training load and disease characteristics. We also tested if differences in FA exist between the different balance sports in group T. For all analyses, whole brain voxelwise statistical analyses were carried out using a Monte Carlo permutation method provided by the Randomise tool in FSL. Note that unless otherwise stated 5000 permutations were used and age was always added as a covariate of no interest in statistical designs. P-value statistical images were fully corrected for multiple comparisons across space and were generated using threshold-free cluster enhancement (TFCE) [19]. All analyses and results were considered significant if they survived the corrected threshold of p<0.05. White matter tracts were specified using the JHU DTI-based white-matter atlases [20] included in FSLView.

#### Age related issues

A major challenge of the statistical analysis of our data was that the two study groups, BVF and T, respectively their control groups, BC and TC, differed notably in age. This was inevitable, as mainly young persons regularly perform balance sports, while bilateral vestibular failure usually occurs at an advanced age. White matter FA shows considerable changes over the lifespan. It increases during childhood and adolescence, reaches a peak during adulthood and from middle age on decreases [21]. This issue made it hard to directly compare the young with the old groups. For our statistical analyses we compensated for this in several ways: Wherever possible, we compared groups that were age-matched. This applies to the separate comparisons of BVF vs. BC and T vs. TC as well as to the combined comparison of BVF+T vs. BC+TC. Additionally, we added age as a covariate in these analyses to avoid any age-related confounds.

However, the direct comparison between the two different study groups remains an interesting topic. We therefore directly compared the two study groups using age as a covariate. As the strong confound of age might overshadow actually present differences between the groups, we additionally conducted an analysis to compare BVF and T, by subtracting age-related effects beforehand. We created new FA “difference” maps reflecting the difference between BVF and BC and T and TC respectively, by subtracting the skeletonised FA map of the age-matched control from the respective study subject FA map. In this way, we created 13 difference maps for BVF - BC and 18 difference maps for T – TC. The missing TC control subject was replaced by the mean FA skeleton of group TC. We then performed the voxelwise statistical analysis on these difference maps.

We further investigated the general effect of age on FA by analyzing the correlation between age and FA. This analysis was performed by adding contrasts investigating the effect of the covariate age to the design matrix containing all subjects of the four groups.

#### Group comparisons

For group comparisons a model was used in which each of the four groups were modeled as a separate column, and age was a regressor of no interest. First, we compared BVF and T separately to their respective control groups. This analysis showed us where patients had FA changes compared to their healthy age- and gender-matched controls and independent of this, where trained individuals significantly differed from their control group. We then tested for differences between study and control groups, by comparing BVF and T, respectively BC and TC, first directly with age as a covariate, then by using the age-matched difference maps of FA.

In a last analysis, we compared BVF and T as a single group, to their control groups. Using the original model from the first analysis, we looked at the difference between the study groups as a whole (BVF+T) compared to the two control groups (BC+TC).

#### Analysis of axial and radial diffusivity

Water diffusion within the white matter of the brain is commonly used as an indicator of fiber integrity. This is because the fatty myelin layers and the cytoskeleton of the nerves determine a principal diffusion direction which is axial, i.e. parallel to the nerve fibers. Consequently, a loss of fiber integrity as well as fiber crossings within a specific region reduce diffusivity along that principal axis and promote perpendicular diffusion directions [12], [22], [23]. To understand the nature of the differences in FA, we calculated RD and AD for all voxels in each subject. FA is calculated in FSL according to formula (1) from the three eigenvalues (λ1,λ2,λ3) that describe the size and shape of the diffusion tensor. One can see that decreases in FA can either be caused by increases in RD, decreases in AD or a combination of the two [23]. FSL output images representing the voxelwise eigenvalues (L1, L2 and L3) were used to calculate the RD and the AD according to the formulas (2) and (3).
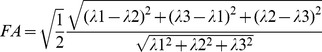
(1)

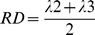
(2)

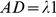
(3)For all analyses showing significant differences in FA, we conducted identical whole brain voxelwise statistical analyses for RD and AD, to identify the driving tensor component that caused the changes in FA. For each analysis, 500 permutations were run.

#### Behavioral analyses

Because of the heterogeneity of the groups tested, we also collected data about cognitive and memory performance from all of the subjects in this study. All subjects performed the MWT-B (“Mehrfachwahl-Wortschatz-Intelligenztest B”) and the Doors A and B subtest of the Doors and People test [24]. The MWT-B is a German-language modification of the National Adult Reading Test of Nelson and measures premorbid intelligence. The Doors test provides an estimate for visual recognition memory asking the subject to memorize a colored door and to identify it in an array of four doors. The two parts of the Doors test each have a maximum score of 12 points, the results of which were averaged into a single test score per subject. The MWT-B had a maximum score of 37. Using a one-way ANOVA with four levels, we compared the group means of the test results to test for significant differences in intelligence or memory between the different groups. Further, we correlated the individual test results with the voxelwise FA values to test for inter-subject interdependencies between FA and cognitive performance. Correlation analyses were performed on the demeaned test values using the Randomise tool in FSL with 500 permutations. For six subjects (1 BVF, 2 T, and 3 TC), no data of the MWT-B test could be collected. For these subjects, the average MWT-B score across all four groups was substituted in place of the missing data.

It is well known that patients suffering from vestibular loss usually show spatial memory deficits [1], and that the virtual Morris Water Task provides useful behavioral measures for spatial memory performance. However, the virtual Morris Water Task must be altered for ageing populations such that a direct comparison of spatial memory performance using this task was not possible [1], [25].

In addition to cognitive and memory performance, we also looked at whether training or disease-related measures correlated with white matter FA values. For the BVF study group, we chose the caloric mean slow phase eye velocity, which is a well-known measure of the nystagmus following vestibular loss, and the time since onset of the disease as disease-related measures. As a measure of training in the T study group, we used the current training load (h/week) and the overall training experience. Training experience was calculated from the weekly training time in hours averaged across the last year multiplied with the number of years since beginning the training [11]. We also tested if differences in FA existed depending on the kind of balance sport (ballet, figure skating or slacklining) the individuals of group T performed by performing a whole brain voxelwise one-way ANOVA across the FA values within the three different subgroups of T. These analyses were done with the Randomise tool in FSL with 500 permutations.

## Results

### 1. Comparisons between study groups and their controls

**Figure 1:**
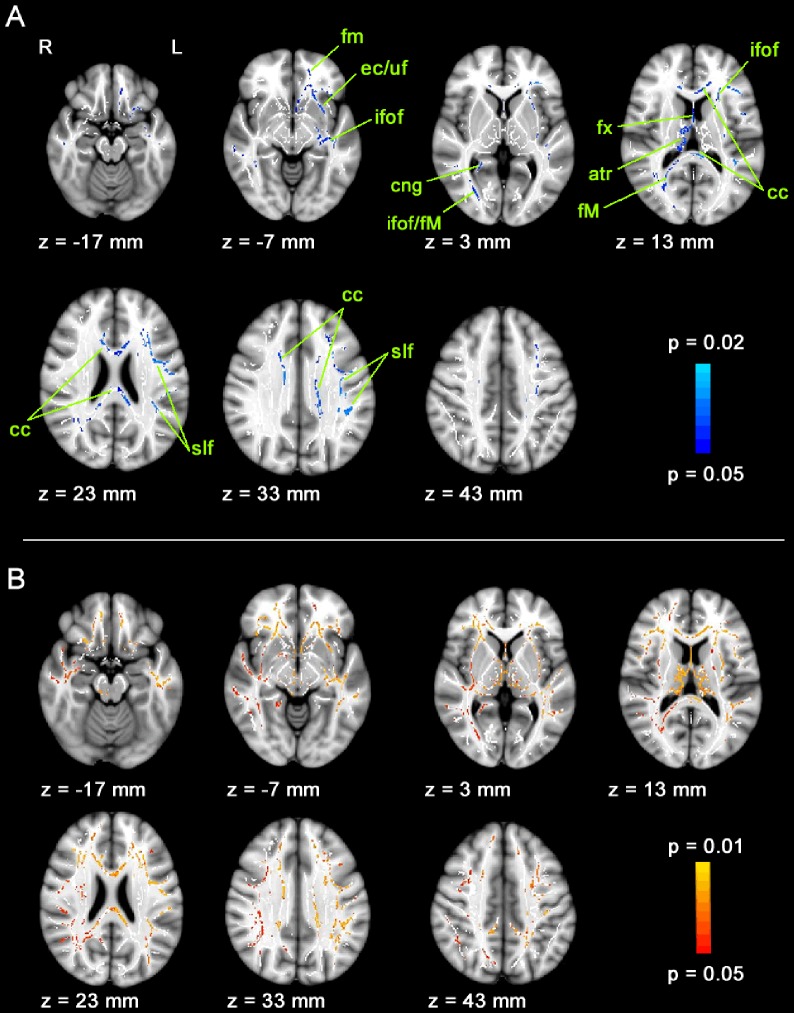
Fractional anisotropy and radial diffusivity changes in patients suffering from vestibular loss. A. FA is reduced in patients compared to their healthy control group. Voxels showing a significant lower FA of BVF compared to BC are shown in blue. Altogether, 11,546 voxels were significant; atr = anterior thalamic radiation, cc = corpus callosum, cng = cingulum, ec = external capsule, fm = forceps minor, fM = forceps major, fx = fornix, ifof = inferior fronto-occipital fasciculus, uf = uncinate fasciculus, slf = superior longitudinal fasciculus. B. RD is higher in patients compared to the control group in similar areas (red-yellow). Significant voxels are overlaid on seven axial slices of the MNI152_T1_1mm_brain standard image included in FSL and the mean FA skeleton mask (white).

Each study group was compared to their respective control group. The comparison between patients and their control group showed that BVF patients had a reduced FA in distributed white matter pathways (Figure 1A). Affected fibers could be found in the corpus callosum, the anterior and posterior forceps, the right anterior thalamic radiation, the fornix, the left external capsule, the left uncinate and superior longitudinal fasciculus and bilaterally in the inferior fronto-occipital fasciculus.

The analysis of the axial and radial diffusion components showed an increase in RD in similar brain regions for BVF compared to BC (Figure 1B), while AD was also slightly but not significantly increased (p = 0.078). This suggests that the reduced FA is a result of a stronger radial diffusion rather than of less axial diffusion.

No significant differences in FA were found between trained subjects and their control group.

### 2. Differences between patients and trained persons

**Figure 2:**
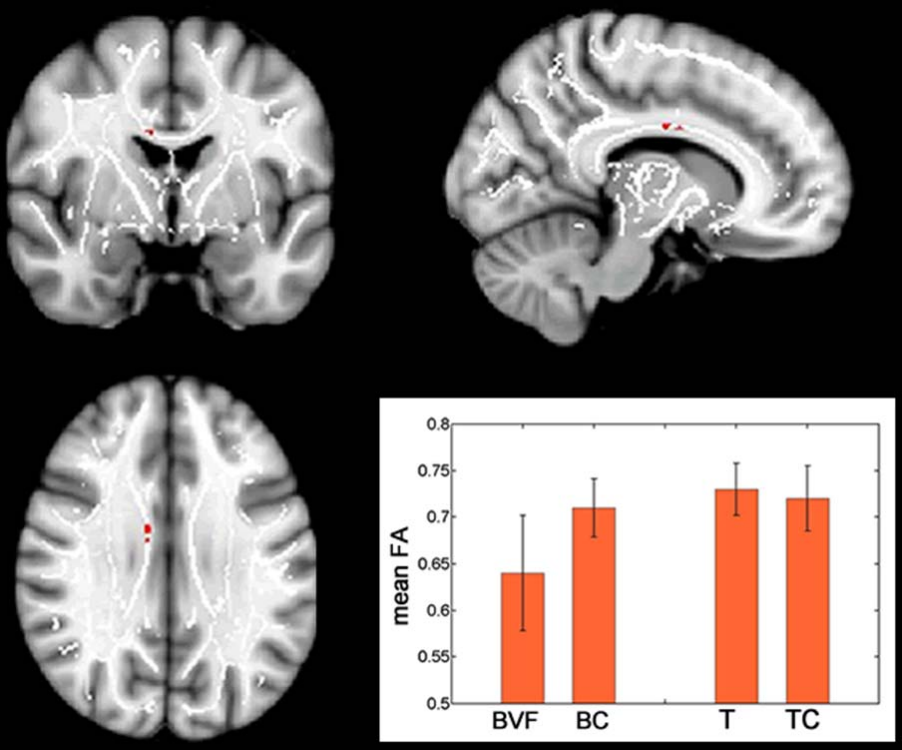
FA difference map comparison. FA difference maps were created and compared for BVF-BC and T-TC. These maps differed in a small area of the corpus callosum (red). Within this area, patients (BVF) had a lower FA compared to their control group (BC), while balance trained persons (T) had a slightly higher FA compared to their control group. Significant voxels are overlaid on the MNI152_T1_1mm_brain standard image (x = 10 mm, y = −3 mm, z = 29 mm) included in FSL and the mean FA skeleton mask (white). The cluster of significant voxels comprised 53 voxels.

No significant differences were found for BVF and T, nor for BC and TC, when comparing them directly, with age as a covariate. In order to compare patients and trained persons without the confound age, we created and compared the FA difference maps of the two study groups and their respective control groups. Significant differences were found only in a small region of the corpus callosum (Figure 2). In this region, the difference in FA was significantly greater between BVF and BC than between T and TC. We then looked at those voxels in the mean FA maps for each group. BVF showed a lower FA in this region than BC, while T had a higher FA than TC. No significant differences were found for the comparison of the RD and AD maps.

### 3. Comparison of both study groups together to their controls (BVF+T vs. BC+TC)

**Figure 3:**
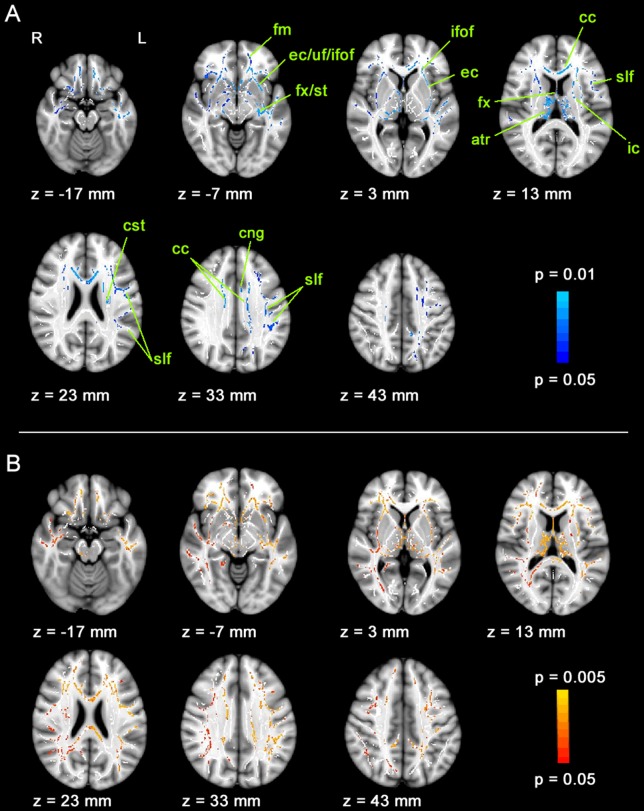
Fractional anisotropy and radial diffusivity changes of patients and balance trained persons. Both study groups, patients with bilateral vestibular loss and balance trained individuals show FA reductions and RD increases compared to their control groups. A. Voxels showing a significant lower FA of BVF+T compared to BC+TC are shown in blue. Altogether, 21,933 voxels were significant; atr = anterior thalamic radiation, cc = corpus callosum, cng = cingulum, cst = corticospinal tract, ec = external capsule, fm = forceps minor, fx = fornix, ic = internal capsule, ifof = inferior fronto-occipital fasciculus, uf = uncinate fasciculus, slf = superior longitudinal fasciculus, st = stria terminalis. B. RD is higher in the study groups compared to the control groups in similar areas (red-yellow). Significant voxels are overlaid on seven axial slices of the MNI152_T1_1mm_brain standard image included in FSL and the mean FA skeleton mask (white).

Patients and balance trained subjects showed similar changes of FA compared to controls in widespread white matter tracts (Figure 3A). All affected brain regions showed a reduction of FA in the study groups compared to the control groups. No regions showed a significant increase of FA. Areas of reduced FA in patients and trained individuals can be sorted into different functional categories. First, the corpus callosum, which is the main connection between the two hemispheres, was affected. Second, the thalamus, which is the main relay station for peripheral fibers running to the cortex, showed reduced FA in its entire white matter skeleton. Then, the internal capsule, carrying fibers of the corticospinal tract, the main motor pathway, showed reduced FA. The fornix, an intralimbic communication center connecting various limbic structures including the hippocampus, the septal region, the mammillary bodies, the prefrontal cortex and the cingulum was also affected. Finally, FA reductions also apply to association fibers. The inferior fronto-occipital, superior longitudinal and uncinate fasciculus, which all connect the frontal lobe to rostral parts of the brain, all had a reduced FA. The analysis of the diffusion components showed, that the increase in FA in these areas is a result of a significantly higher RD in the two study groups compared to the control groups (Figure 3B). AD was also slightly, but not significantly increased. Mean FA, RD and AD within significant voxels are summarized in Table 3.

**Table 3:** Mean FA, RD and AD (± SD) across all voxels that survived thresholding for the comparison between study groups and control groups.

	FA	RD (* 10^−4^)	AD (* 10^−3^)
**BVF**	0.45±0.029	6.61±0.72	1.35±0.056
**T**	0.50±0.0082	6.61±0.19	1.54±0.040
Ballet	0.50±0.0081	6.59±0.28	1.53±0.023
Figure skating	0.49±0.0064	6.65±0.15	1.53±0.041
Slacklining	0.50±0.0075	6.62±0.22	1.55±0.049
**BC**	0.49±0.017	5.86±0.29	1.32±0.030
**TC**	0.52±0.014	6.30±0.26	1.53±0.046

### 4. Effects of age on FA

**Figure 4:**
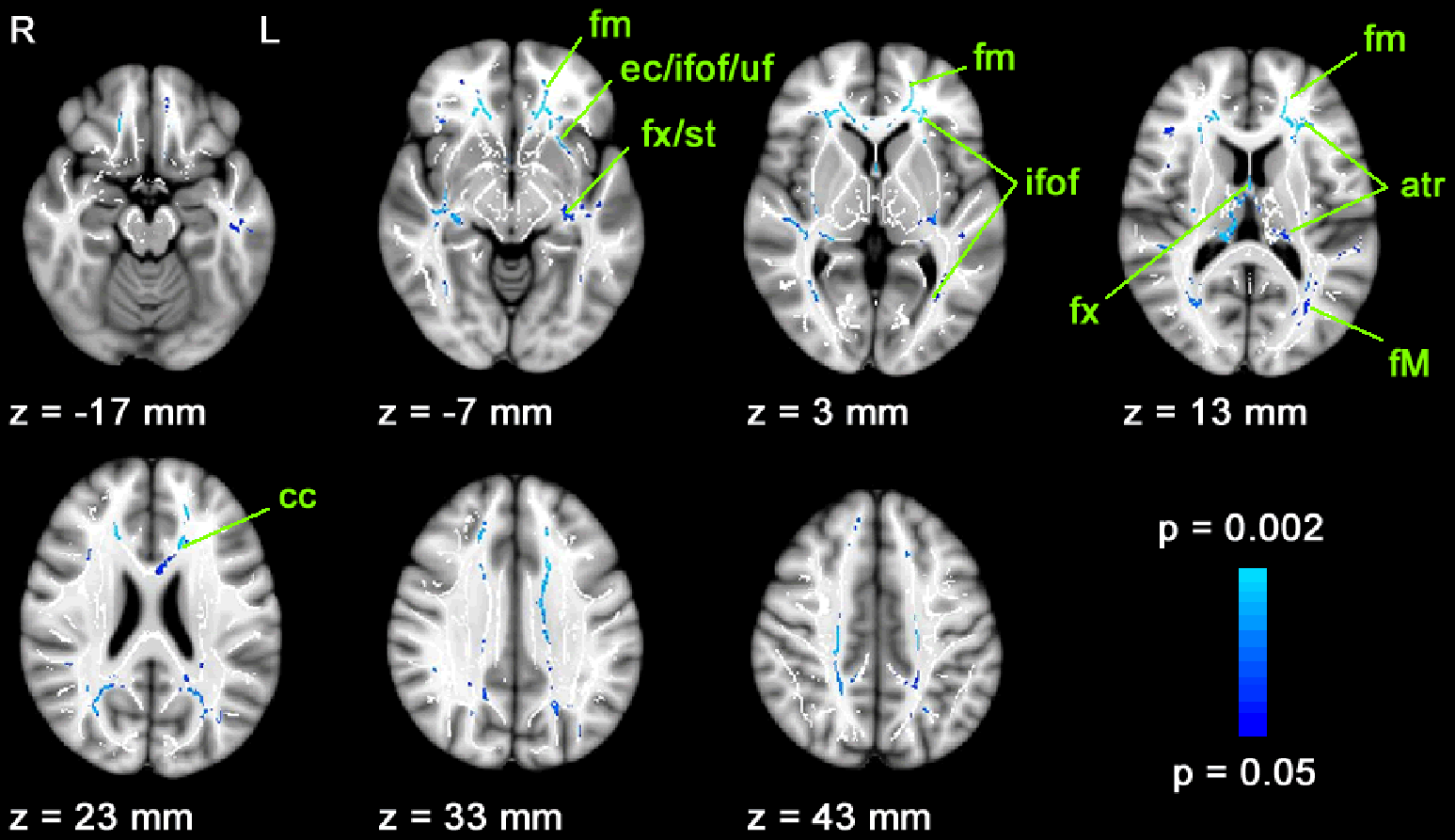
Fractional anisotropy correlates with age. FA values decrease with increasing age in widespread areas of white matter tracts. WM tracts showing significant correlation between FA and age of all 61 subjects are shown in blue. Altogether, 12,868 voxels were significant; atr = anterior thalamic radiation, cc = corpus callosum, ec = external capsule, fm = forceps minor, fM = forceps major, fx = fornix, ifof = inferior fronto-occipital fasciculus, uf = uncinate fasciculus, st = stria terminalis. Significant voxels are overlaid on seven axial slices of the MNI152_T1_1mm_brain standard image included in FSL and the mean FA skeleton mask (white).

Across all subjects of the four groups, FA showed a significant negative correlation with age in wide-spread white matter regions (Figure 4). Interhemispheral connections, i.e. fibers of corpus callosum, forceps minor and forceps major, connective fibers between thalamus and frontal cortex (anterior thalamic radiation) as well as between thalamus and visual cortex (optic radiation), the fornix of the limbic system and association fibers of the uncinate and inferior-occipital fasciculus showed significant FA decreases with age.

### 5. Behavior and FA changes

To test for cognitive differences in the study groups, all participants performed the MWT-B and the Doors test. The one-way ANOVA showed significant differences between the groups on both tests (MWT-B: F(60) = 3.89, p = 0.013, Doors: F(60) = 3.02, p = 0.037). The results are shown in Table 4. No single group showed systematic performance on the cognitive tests. Mean MWT-B scores (± SD) were 32.45 (±2.58) for BVF, 31.77 (±2.71) for BC, 28.82 (±4.64) for T and 31.15 (±1.81) for TC out of a total score of 37, and the mean Doors test scores were 8.42 (±2.14) for BVF, 9.15 (±1.34) for BC, 9.75 (±1.51) for T and 10.05 (±1.30) for TC out of a total score of 12. Interestingly, the patients performed better on the MWT-B test than trained subjects, whereas TC performed better on the Doors test than the patients.

**Table 4:** Results of the cognitive performance tests.

ID	Doors	MWTB	ID	Doors	MWTB
BVF01	5.5	N/A	BC01	10	31
BVF02	7.5	32	BC02	8	35
BVF03	9	33	BC03	10	34
BVF04	9.5	34	BC04	9.5	26
BVF05	6.5	30	BC05	11.5	29
BVF06	11.5	36	BC06	8	31
BVF07	10	29	BC07	8	30
BVF08	9	36	BC08	11.5	33
BVF09	11.5	32	BC09	9	35
BVF10	10.5	35	BC10	9	29
BVF11	6.5	35	BC11	7	33
BVF12	7	30	BC12	8.5	33
BVF13	5.5	29	BC13	9	34
T01	10	34	TC01	11.5	30
T02	8.5	N/A	TC02	8.5	33
T03	11.5	22	TC03	9.5	30
T04	11	33	TC04	9.425	N/A
T05	10.5	28	TC05	8	N/A
T06	9.5	27	TC06	7.5	34
T07	9.5	23	TC07	9.5	31
T08	7.5	19	TC08	10	30
T09	11.5	26	TC09	11.5	32
T10	11.5	27	TC10	10	33
T11	8	25	TC11	9.5	28
T12	10	34	TC12	12	32
T13	12	34	TC13	11.5	27
T14	6.5	34	TC14	10	32
T15	10	31	TC15	10.5	32
T16	8.5	N/A	TC16	10.5	33
T17	9.5	27	TC17	11.5	N/A
T18	10	33			

Values represent the amount of correct responses out of 12 possible responses for the Doors and out of 37 possible responses for the MWT-B test.

We also correlated the test scores of the cognitive tests with the voxelwise FA values for each subject, to test if the neuropsychological tests correlated with white matter diffusivity. However, no significant correlations between cognitive performance and FA values were found, suggesting that the results that we do see, are not related to cognitive performance.

We also assessed whether disease- or training-related measures were correlated with differences in FA. We did not find significant differences in FA between the different balance sport types ballet, figure skating and slacklining within the trained group. Further, current training load and training experience did not correlate with the FA. Also, patients' FA did not correlate significantly with the caloric mean slow phase eye velocity or the time since onset of the disease. Taken together, these results suggest an overall effect of balance effort on FA changes instead of specific training or disease-related effects.

## Discussion

The separate comparison of our study groups versus their respective control groups shows significant FA reductions for patients compared to their healthy control individuals, while FA reductions for trained subjects compared to their control group are not significant. Considering the small effect sizes and large number of statistical tests performed, we cannot conclude from our findings that no difference in FA exists between balance trained individuals and their controls. The mean FA over the entire white matter tract of T was lower than that of TC, which was also reflected in the comparison of both study groups together to the two control groups. This suggests, that the reductions in FA in balance-trained individuals, although not significant, affect the same white matter tracts than the FA reductions caused by bilateral vestibular failure. These tracts include widespread sensory, motor, limbic and association pathways.

Hänggi et al [10] found significantly reduced FA as well as changes in white- and gray-matter volume in professional ballet dancers. Although in our study the reduction of FA for balance trained individuals was not significant, we believe that our results are consistent with this study. Hänggi et al. tested only young female ballet dancers between 18 and 25 years, who had been training for 14.2±3.3 years. Our test subjects were female and male ballet dancers, figure skaters and slackliners between 16 and 43 years, whose total training period ranged from 1 to 34 years. We believe that the differences in groups, and in particular the heterogeneity in our trained group makes direct comparisons between the two studies difficult. However, the combined analysis of both study groups compared to the control groups shows reduced FA in areas that overlap with those found in the Hänggi et al. study.

The direct comparison of T and BVF was partially confounded by the age differences between patient population and balance-trained individuals. Using age as a covariate for the comparison of BVF and T respectively of BC and TC, we found no significant changes in FA between the groups. The negative correlation between age and FA in our data affects a broad network of white matter fibers, consistent with the literature on age-related changes in FA [21], [26]. Still, using difference maps to subtract out possible age-related effects, differences between study groups were limited to a small area within the corpus callosum. Here patients showed a lower FA than their controls, while trained subjects show a higher FA than their controls. The corpus callosum is involved in a wide range of processes and connects primary and secondary motor areas between the two hemispheres [27] and as such may represent a real effect of increased vestibular training that is then decreased with less vestibular input. However, the nature of the analysis done can artificially inflate spurious differences between individuals; therefore these results should be regarded with skepticism before they are confirmed by future work.

Taken together, we conclude from our findings that both, balance training and bilateral vestibular failure cause a decrease of white matter FA that affects very similar white matter tracts in the brain. Within affected white matter tracts we find a significant increase of the radial diffusivity component. Thus, the reductions in FA in our study groups were likely a result of an increase in water diffusivity along the perpendicular diffusion directions, and not a decrease in the diffusivity along the main direction of water diffusion. They were not correlated with measures of intelligence, memory, training load or characteristics of disease, and they existed independent of the age difference between the two study groups and independent of the kind of balance sport that the individuals of group T performed.

Why is it that patients with vestibular loss, who have severe problems maintaining balance, show the same pattern of white matter plasticity as subjects who regularly perform balance sports and can maintain balance in even the most difficult of situations? We cannot exclude that we are looking at separate but overlapping effects. For a defective functioning of a sensory system, a reduction of fiber integrity seems plausible and has been seen in the past [28]–[30]. In contrast, it is not likely that healthy balance trained subjects show a pathologically induced loss of fiber integrity in these same regions, but is more likely due to crossing fibers [10]. Because the changes in FA that we found did not correlate with disease characteristics, the changes seen here may not be directly related to the pathology of the disease. Diffusion spectrum imaging together with q-ball imaging [31] where a more complicated model for diffusion is used, may help to differentiate between increasing crossing fibers, and a reduction in fiber integrity.

Alternatively, the highly similar changes in FA and RD across both study groups rather suggest common underlying causes. The most striking behavioral characteristic that T and BVF have in common compared to their control groups is the increased effort that they have to make in order to maintain balance. Both groups need to increase balance beyond the level that is usually needed or can be guaranteed by the available sensory input under normal circumstances [32]–[34]. To avoid imbalance and falls, patients suffering from bilateral vestibular failure must make an effort, in part by using other sensory inputs, to compensate for the missing vestibular information. Individuals of group T do not have problems in maintaining balance under normal conditions, but during training they too must use all available sensory inputs to maximize balance and reduce vertigo. The compensation process that follows vestibular loss [35] may be the key to the structural reorganization of white matter tracts involved in balance maintenance.

An alternative common mechanism for the decreased FA in both study groups is that they both might need to suppress or reinterpret vestibular input to maintain balance. Ballet dancers reduce vestibular responses to increase balance during a pirouette [5]. When a slackliner balances on the shaking rope, typical postural responses would induce compensatory movements that could cause falls. A suppression of the vestibular information and an enhancement of visual and proprioceptive input instead would be beneficial in these cases, and have been seen for these populations [5], [7]. For patients who suffer from bilateral vestibular failure, the vestibular input is reduced or non-existent. Thus, the effects on white matter could be similar to the effects in balance trained persons who suppress vestibular responses. It is even possible that a defective rest vestibular input remains in patients [36]. In this case, a suppression or reinterpretation of this vestibular input would help to maintain balance [11], particularly after compensation has occurred.

Central vestibular processing is spread across multiple brain regions, that integrate multimodal information [37]. The extent to which short-range as well as long-range white matter fibers were affected in our study is consistent with the multimodal nature of cerebral vestibular processing. The entire thalamus was affected, suggesting the affliction of both bottom-up and top-down pathways, including sensory and motor fibers. The corpus callosum is involved in a wide range of processes and connects primary and secondary motor areas between the two hemispheres, and as such is important for the coordination of movements [27]. That both of these structures were affected suggests that the sensori-motor system was different between the study groups and their controls. The association fiber bundles that were affected play a role in various cognitive processes, e.g. visuospatial processing, object recognition and memory [38], [39]. The long-range connection fibers are further an important messenger between different cortical areas.

The changes in the limbic system are particularly interesting with respect to the hippocampal atrophy and related spatial memory deficits seen in patients with BVF [1]: Vestibular failure is known to be associated with an increase in the level of glucocorticoids [40], [41] and a reductive effect of these hormones on hippocampal volume was shown in various neuropsychiatric diseases [42]. Balance trained individuals have also shown a decrease in the anterior portion of the hippocampus [11], which is often related to emotional and chemical processing. The decrease in FA within the limbic system might therefore relate to a change in relative levels of stress hormones released in these individuals, and warrants further investigation.

Although our data does not provide a definitive explanation for the similarities in white matter structure between BVF patients and ballet dancers, figure skaters and slackliners, we can conclude that bilateral vestibular loss and extensive balance training induce changes in similar white matter tracts. Balance training after vestibular loss is therefore very likely to have an effect on white matter plasticity that could help the coordination of different sensory systems for balance and postural control, and as such may represent a physiological mechanism for balance training as a method of rehabilitation.
